# Effects of anti-aging interventions on intestinal microbiota

**DOI:** 10.1080/19490976.2021.1994835

**Published:** 2021-11-08

**Authors:** Yanjiao Du, Yue Gao, Bo Zeng, Xiaolan Fan, Deying Yang, Mingyao Yang

**Affiliations:** Animal Genetic Resources Exploration and Innovation Key Laboratory of Sichuan Province, Sichuan Agricultural University, Chengdu, Sichuan, China

**Keywords:** Intestinal microbiota, aging, dietary intervention, exercise, drugs, bacteriotherapy

## Abstract

Identifying ways to deal with the challenges presented by aging is an urgent task, as we are facing an aging society. External factors such as diet, exercise and drug therapy have proven to be major elements in controlling healthy aging and prolonging life expectancy. More recently, the intestinal microbiota has also become a key factor in the anti-aging process. As the intestinal microbiota changes with aging, an imbalance in intestinal microorganisms can lead to many age-related degenerative diseases and unhealthy aging. This paper reviews recent research progress on the relationship between intestinal microorganisms and anti-aging effects, focusing on the changes and beneficial effects of intestinal microorganisms under dietary intervention, exercise and drug intervention. In addition, bacteriotherapy has been used to prevent frailty and unhealthy aging. Most of these anti-aging approaches improve the aging process and age-related diseases by regulating the homeostasis of intestinal flora and promoting a healthy intestinal environment. Intervention practices based on intestinal microorganisms show great potential in the field of anti-aging medicine.

## 1. Introduction

Aging is a natural, time-dependent physiological process that results in a decline in overall function. This decline is the primary risk factor for major human pathologies, including cancer, diabetes, cardiovascular disorders, and neurodegenerative diseases.^1^ Reducing the negative impacts of advanced age and increasing healthspan has therefore been an important goal of aging and anti-aging research. Genomic instability, telomere attrition, epigenetic alterations, loss of proteostasis, deregulated nutrient sensing, mitochondrial dysfunction, cellular senescence, stem cell exhaustion, and altered intercellular communication have been proposed as the main molecular and cellular hallmarks of aging.^1^ Some anti-aging interventions can be found by targeting these characteristics and the pathogenesis of aging.^2^ Various anti-aging interventions have been demonstrated to extend the lifespan of model organisms. These interventions are generally classified as dietary intervention, exercise, drug treatment, and genetic alteration.^3^ A large array of genetic alterations have been found to increase lifespan in some model organisms. For example, increasing the sirtuin level through genetic manipulation extends the lifespan of yeast, nematodes and flies.^4^ Reducing mTOR expression can prolong model animals’ lifespan and healthspan.^5^ However, the inability to control gene change hinders its application in human society. Therefore, nongenetic interventions are the focus of current anti-aging research. Dietary intervention, exercise and drug treatment are the most promising interventions for aging and age-related diseases and are included in the research of the US National Institute on Aging.^6,7^

**Figure 1.**
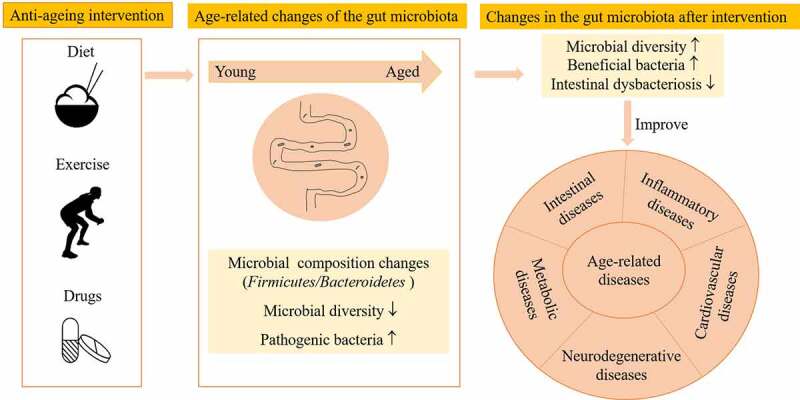
Anti-aging interventions alter the gut microbiota and slow the progression of age-related diseases

The intestine is a critical target organ for improving the health of aged animals and humans.^8^ The intestinal tract harbors an extremely complex and diverse community of microorganisms known as the gut microbiota. The gut microbiota influences the long-term homeostasis of metazoans by maintaining epithelial integrity in the intestinal tract, supporting digestion, training the intestinal immune system, and preventing the growth of pathogenic bacteria.^9^ Abnormal shifts in the gut microbiota are associated with age-related chronic diseases. The gut microbiome is becoming a key factor in the aging process.^10^ Therefore, we speculate that the gut microbiota could be a new anti-aging target. In this review, we will focus on nongenetic interventions, discuss the changes in and beneficial effects of the gut microbiota under various anti-aging approaches and explore the interactions between anti-aging interventions and the gut microbiota (Figure 1).

## 2. Aging and gut microbes

Numerous studies in animals and humans have shown that the composition of the gut microbiota varies with host age. The intestinal microbiota in fruit flies,^11^ fish,^12^ mice,^13–17^ rats,^18^ and humans^19–23^ all undergo age-related changes, most of which are disadvantageous to health (Table 1). The gut microbiota of adult humans is dominated by the *Firmicutes* and *Bacteroidetes* and smaller proportions of *Proteobacteria, Actinobacteria* and *Verrucomicrobia*.^24^ Some studies reported the enrichment of *Bacteroidetes* and *Protobacteria* abundances and a decrease in *Firmicutes* and *Bifidobacteria* in aged people.^19–21^ A recent study has suggested the presence of a different core microbiota dominated by *Ruminococcaceae, Lachnospiraceae* and *Bacteridaceae* families, which decrease with aging.^22^ This difference may be due to significant variability between individuals and external factors. However, long-living people have a unique intestinal microbiota. As shown in Table 1, health-related genera such as *Akkermansia, Bifidobacterium, Christensenellaceae* and microbial diversity are enriched in long-living people.^22,23^ Despite changes in the microbes of long-living people, their diversity and beneficial microbes are preserved to support healthy aging.^25^ Overall, the composition of gut microbes is changed and microbial diversity is reduced due to the accumulation of potentially proinflammatory microbes and a decrease in beneficial microbes with aging.

**Table 1.** The gut microbiota changes with aging

Study Model	Gut Microbiota Variations Induced by Aging	Effects on Health	References
*Drosophila melanogaster*	*Gammaproteobacteria*↑	Impairs intestinal function and drives mortality	Clark, 2015^11^
Turquoise killifish	Microbial diversity↓ Over-representation of pathogenic *Proteobacteria*↑	Leads to pathological gut environment	Smith, 2017^12^
Mice	*Rikenellaceae* family↑	Affects the bioavailability of B vitamins and other metabolites	Langille, 2014^13^
Mice	*Bacteroidetes, Tenericutes*↓ *Firmicutes, Actinobacteria*↑	Increases systemic endotoxin levels	Kim, 2016^14^
Mice	*Firmicutes/Bacteroidetes*↑	Leads to an alteration in metabolism	Vemuri, 2018^15^
Mice	*Firmicutes* (3–14 M↑, 20 M↓) *Bacteroidetes* (3–14 M↓, 20 M↑) *Proteobacteria*↑	Causes a shifts in metabolomic profiles	Luo, 2020^16^
Mice	*Firmicutes/Bacteroidetes*↑	May be involved in the production of some pro-inflammatory metabolites	Piao, 2020^17^
Rats	*Firmicutes/Bacteroidetes*↓	N/A	Flemer, 2017^18^
Humans	*Bacteroidetes*↑	N/A	Claesson, 2011^19^
Humans	*Firmicutes, Bifidobacteria*↓ *Enterobacteriaceae, Bacteroidetes*↑	Impairs health	Rondanelli, 2015^20^
Humans	*Firmicutes* ↓ *Bacteroidetes, Proteobacteria* ↑	N/A	Odamaki, 2016^21^
Humans	Core microbiota ↓ (*Ruminococcaceae, Lachnospiraceae, Bacteroidaceae*) Subdominant species↑	N/A	Biagi, 2016^22^
Humans (≥ 90 years old)	Microbial diversity↑ Several potentially beneficial bacterial taxa ↑ (*Clostridium cluster XIVa, Ruminococcaceae, Akkermansia, Christensenellaceae*)	N/A	Kong, 2016^23^

(M, month; ↑, increase; ↓, decrease; N/A, the effects of changes in gut microbes on health were not mentioned)

Conversely, this age-related disorder of gut microbes can affect the health and longevity of the host.^26^ In fruit flies and mice, age-related microbiota disorders could lead to intestinal barrier dysfunction, which is a pathophysiological marker of aging.^11,27^ Compared with fruit flies fed a homogenate of young flies, those fed homogenized old flies had a shorter lifespan and a higher incidence of intestinal barrier dysfunction.^11^ Transplantation of feces from young donors into the intestines of middle-aged fish can extend the lifespan and delay behavioral decline.^12^ In addition, the genetic composition and metabolites of microorganisms can also have a positive impact on the longevity of the host.^28^ Therefore, gut microorganisms may play a regulatory role in the process of aging.

## 3. Anti-aging interventions and intestinal microbes

Many interventions can be used to regulate aging. Intervention that affects intestinal microbes is an anti-aging strategy. Accordingly, intestinal microbes can be influenced by many anti-aging factors, such as diet, exercise, and drugs.

### 3.1 Diet intervention and intestinal microbes

Diet and dietary patterns have a major role in the pathogenesis of many age-related diseases. Calorie restriction (CR), also known as dietary restriction (DR), is a dietary plan that reduces calorie intake without resulting in malnutrition or reductions in essential nutrients.^29^ Subsequently, new CR schemes, such as intermittent fasting (IF), have been explored. CR can promote corresponding changes in intestinal microflora, so intestinal microflora may play a main role in the beneficial effect of CR. In addition to typical CR measures, many natural foods and healthy dietary habits also have anti-aging effects. Food components and dietary habits can modulate gut microbiota composition and intestinal barrier functions.^30^

#### 3.1.1 CR and IF

CR can extend the lifespan in many species, from invertebrates to rodents and even some nonhuman primates.^31–33^ For a long time, CR has been recognized as one of the most effective nongenetic dietary interventions that can increase lifespan and prevent age-related diseases.^32,33^ CR/DR can also result in changes in the intestinal microbiota and metabolome. These CR-induced changes in the gut microbiota suggest that animals can manage CR to establish a balanced composition of the gut microbiota, which provides a health advantage to the host.

In normal animals, CR treatment enables changes in the microbiota structure, reduction in diet-associated metabolic disorders and a prolonged and healthy lifespan. *Lactobacillus* was significantly increased after CR treatment for 8 weeks, and its relative abundance was significantly higher than that of randomly fed rats after 36 weeks.^34^ In mice, CR significantly altered the overall structure of the intestinal microbiota, enriched the phyla positively associated with longevity (such as *Lactobacillus and Bifidobacterium*), and reduced the phyla negatively associated with longevity.^35–37^ Moreover, short-term CR was sufficient to significantly restore intestinal microbiota imbalance to a more balanced state, as seen in younger mice, by reducing the dominance of *Clostridia, Clostridiales*, and *Firmicutes*.^38^ However, the duration of CR can affect changes in host metabolic phenotypes and intestinal microbiota. The intestinal microbiota of light-fed CR mice was significantly different from that of dark-fed CR and random-fed mice.^39^

In obesity models, CR leads to relevant changes in gut microbiota that counteract the metabolic damage associated with obesity and a high-fat diet. Forty-five days of CR in obese mice enriched *Bacteroidetes* and significantly reduced the *Firmicutes:Bacteroidetes* ratio.^40^ CR treatment also causes similar microbial changes in humans. Long-term (one year) CR in obese adolescents also reduced the *Firmicutes:Bacteroidetes* ratio and enhanced the growth of beneficial microorganisms such as *Bacteroides, Roseburia, Faecalibacterium* and *Clostridium*.^41^ However, a very-low-calorie diet (VLCD) reduced the number of *Bacteroides* and increased *Firmicutes* in obese patients.^42^ VLCD led to a decrease in bacterial abundance and restructuring of the gut microbiome in overweight or obese women. Transplantation of post-diet microbiota to mice decreased their body weight and was enriched with the enteric pathogen *Clostridioides difficile*.^43^

IF is a type of periodic CR that has also been shown to improve metabolism by reducing body weight and fat mass, lowering blood glucose, and improving insulin sensitivity.^44^ IF is usually achieved by limiting eating for 12–24 hours, and the time interval between fasting may affect the effectiveness of IF. IF could alter the gut microbiota, and the effect was more pronounced in mice that fasted for 16 hours a day; however, these effects disappeared after fasting stopped.^45^

IF can increase the diversity of gut microbes and change their composition, resulting in a higher abundance of *Lactobacillaceae, Bacteroidaceae* and *Prevotellaceae*. Moreover, the transplantation of IF mouse microbiota to normally fed mice improved experimental autoimmune encephalomyelitis, suggesting that the immunomodulatory effect of IF is at least partially mediated by intestinal microbiota.^46^ In mouse models of inflammatory bowel disease (IBD), a fasting-mimicking diet can promote intestinal regeneration, thereby improving IBD-related phenotypes and promoting the expansion of the beneficial intestinal flora *Lactobacillaceae* and *Bifidobacteriaceae*.^47^ Islamic fasting, which is similar to IF, leads to an increase in *Akkermansia muciniphila* and *Bacteroides fragilis* groups, which are considered to be healthy gut microbiota.^48^

IF is an effective and natural strategy for weight control. An every-other-day fasting (EODF) regimen significantly improved obesity, insulin resistance, and hepatic steatosis. Transplantation of microbiota from EODF-treated mice to germ-free mice improved metabolic homeostasis.^49^ EODF for 7 months produced microbiota reconfiguration in diabetic mice, leading to the enrichment of *Firmicutes* and a reduction in *Bacteroidetes* and *Verrucomicrobia*, promoting the integrity of the intestinal barrier.^50^ The 28-day IF regimen for diabetic mice improved behavioral impairment via the microbiota-metabolite-brain axis. Moreover, this regimen improved intestinal barrier integrity and microbial diversity in diabetic mice and increased *Lactobacillus* and butyrate-producing *Odoribacter* while decreasing *Enterococcus, Streptococcus*, and unknown *Enterococcaceae*.^51^ In addition, EODF prevented the development of hypertension in a spontaneously hypertensive stroke-prone rat model, and this effect was mediated by alteration of the gut microbiota.^52^

#### 3.1.2 Natural food products and healthy dietary habits

With the increase in evidence directly linking diet and health, several plants and plant extracts (e.g., fruit extracts, leaf extracts, root and tuber extracts) have emerged as possessing potential health benefits. A healthy diet is considered to be rich in fruits, vegetables and drinks, with these foods being strongly associated with overall wellbeing mainly due to the presence of phenolic compounds and fiber.^53^ Polyphenols and fiber, two of the most important plant constituents, have both been studied regarding their microbiota-modulating potential. Several polyphenols were identified to promote the growth of healthy intestinal microflora (e.g., *Bifidobacterium, Lactobacillus, Akkermansia, Christensenellaceae*, and *Verrucomicrobia*) and have potential anti-aging effects.^54^ For example, the abundance of the intestinal microbiota that may be associated with aging was limited by the intake of lemon polyphenols.^55^ Dietary consumption of anthocyanins increased *Bacteroidetes* and short-chain fatty acids (SCFAs) and decreased *Firmicutes*.^56^ Red wine polyphenols significantly increased the number of *Bifidobacteria* and *Lactobacillus* and butyrate-producing bacteria (*Faecalibacterium prausnitzii* and *Roseburia*) but decreased undesirable bacterial groups such as *Escherichia coli* and *Enterobacter cloacae*.^57^ A green tea and black tea polyphenol diet reduced body weight, resulting in decreased *Firmicutes* and increased *Bacteroidetes* in the cecum.^58^ Dietary fiber, which leads to the production of key metabolites such as SCFAs (beneficial to health), have the potential to change the gut microbiota and alter metabolic regulation. Although current studies have shown that the effects may be associated with an increased abundance of SCFA producers and alterations in microbiota diversity, the interpretation is complicated due to methodological differences.^59^

Healthy eating habits, such as the Mediterranean diet (MD), can also influence gut microbiota. MD, centered on fruits, vegetables, olive oil, nuts, legumes, and whole grains, has been linked to a large number of health benefits.^60^ Better adherence to the MD was associated with significantly higher levels of total SCFAs.^61^ Participants with a high adherence to the MD had lower *Escherichia coli* counts and a higher *Bifidobacteria:E. coli* ratio.^62^ These findings demonstrated the relationship between MD and improvements in the diversity and richness of gut microbiota. Modifying dietary habits and adopting MD may be solutions to prevent microbiota disorders and many gastrointestinal and neurological disorders.

*Firmicutes, Bacteroidetes* and *Proteobacteria* are the main phyla among the intestinal microorganisms in mammals. Dietary intervention led to significant changes in the main phylum or low-level classification group (Table 2). These changes in the relative abundance of bacteria are not always consistent, possibly due to different genetic backgrounds, diets and time restrictions. However, under diet intervention, the main trend of intestinal microorganisms is the increase in beneficial microorganisms, such as *Lactobacillus, Bifidobacterium*, and butyrate-producing bacteria. As such, CR and IF, adding extracts rich in potentially bioactive compounds and maintaining good dietary patterns may bring some additional benefits to improving overall health and wellbeing. The effect of diet on aging is a complex topic. In addition to the above, there are some other anti-aging dietary components and types, such as vitamins, trace elements, protein restriction, and the dietary pattern of Okinawan people, which are not elaborated here.

**Table 2.** Gut microbiota variations induced by dietary regimen

Study Model	Dietary Regimen	Gut Microbiota Variations Induced by Dietary Regimen	Effects on Health	References
Rats	8-week 30% CR	*Lactobacillus*↑	Reduces cholesterol and triglyceride levels	Fraumene, 2018^34^
Mice	62-week 30% CR	*Lactobacillus*↑	Prevents HFD-induced obesity and promotes liver health	Zhou, 2012^35^
Mice	Lifelong 30% CR	*Lactococcus*↓ *Lactobacillus, Bifidobacterium*↑	Reduces serum levels of LPS-binding protein	Zhang, 2013^36^
Mice	30% CR	*Helicobacter pylori*↓ *Lactobacillus, Bifidobacterium*↑	Reduces body weight and alleviates hepatic lipid accumulation	Wang, 2018^37^
Mice	2-month 30% CR	*Clostridia, Clostridiales, Firmicutes*↓	Reduces body weight and fat accumulation	Zeng, 2019^38^
Obese mice	45-day 25% CR	*Firmicutes/Bacteroidetes*↓ *Bacteroidetes*↑	Improves lipid profile and decreases blood glucose level	Russo, 2016^40^
Obese adolescents	1 year 30% CR	*Firmicutes/Bacteroidetes*↓ *Bacteroides, Roseburia, Faecalibacterium, Clostridium*↑	Reduces plasma insulin levels	Ruiz, 2017^41^
Obese people	VLCD (800 kcal/day)	*Bacteroides*↓ *Firmicutes*, butyrate-producing bacteria↑	Reduces body weight	Damms-Machado, 2015^42^
Overweight or obese women	VLCD (800 kcal/day)	*Roseburia, Ruminococcus, Eubacterium*↓ *Akkermansia*↑	Improves metabolic health and reduces body weight	Von Schwartzenberg, 2021^43^
Mice	1 month 16 h fasting per day	*Ruminococcaceae, Alistipes*↓ *Akkermansia*↑	Improves metabolic health	Li, 2020^45^
Multiple sclerosis mice	EODF	*Lactobacillaceae, Bacteroidaceae, Prevotellaceae*↑	Enhances antioxidative pathways	Cignarella, 2018^46^
IBD mice	4-day Fasting-Mimicking diet	*Lactobacillaceae, Bifidobacteriaceae*↑	Partially reverses intestinal inflammation	Rangan, 2019^47^
Humans	Islamic fasting (17 h of fasting/day during a 29-day period)	*Akkermansia muciniphila, Bacteroides fragilis*↑	Reduces total cholesterol and fasting glucose levels	Ozkul, 2019^48^
Mice	EODF regimen	*Firmicutes*↑	Improves obesity, insulin resistance and hepatic steatosis	Li, 2017^49^
Diabetic mice	7-month EODF regimen	*Firmicutes*↑ *Bacteroidetes, Verrucomicrobia*↓	Promotes the integrity of the intestinal barrier	Beli, 2018^50^
Diabetic mice	28-day IF regimen	*Enterococcus, Streptococcus, unknown Enterococcaceae*↓ *Lactobacillus*, butyrate-producing *Odoribacter*↑	Improves behavioral impairment	Liu, 2020^51^
Spontaneously hypertensive stroke-prone rats	EODF regimen	*Proteobacteria*↓	Reduces blood pressure	Shi, 2021^52^
Mice	Lemon polyphenols	*Firmicutes/Bacteroidetes*↓	Delays aging and locomotor atrophy.	Shimizu, 2019^55^
Mice, rats, rabbits	Anthocyanins	*Firmicutes*↓ *Bacteroidetes*↑	Improves intestinal parameters	Verediano, 2021^56^
Obese patients	Red wine polyphenols	*Escherichia coli, Enterobacter cloacae*↓ *Bifidobacteria, Lactobacillus*, butyrate-producing bacteria (*Faecalibacterium prausnitzii, Roseburia*)↑	Reduces markers of the metabolic syndrome	Moreno-Indias, 2016
Obese mice	Tea polyphenol	*Firmicutes*↓ *Bacteroidetes*↑	Reduces body weight	Henning, 2018^58^
Humans	Dietary fiber	SCFA-producers, microbiota diversity↑	Alters metabolic regulation	Myhrstad, 2020^59^
Humans	Mediterranean diet	*Escherichia coli*↓ *Bifidobacteria:E. coli ratio*↑	Improves intestinal health	Mitsou, 2017^62^

(↑, increase; ↓, decrease)

### 3.2 Exercise and intestinal microbes

A sedentary lifestyle has been linked to higher rates of chronic diseases such as cardiovascular disease, cancer and diabetes. Exercise is a powerful preventive and therapeutic intervention that can effectively prevent multiple chronic diseases and improve quality of life.^63^ Many studies have shown that exercise is associated with intestinal microbiota and that exercise-induced changes in intestinal microbiota may have an impact on intestinal and systemic health.

#### 3.2.1 Changes in intestinal microbes caused by exercise

Exercise is often effective in preventing the onset of obesity, and in the process, gut microbes will change accordingly. An increase in butyric acid-producing bacteria affects the metabolic pathway of fat accumulation and prevents obesity.^64^ Several studies have shown that exercise training increased the relative abundance of butyrate-producing taxa, such as *Bacteroidales* S24-7, *Clostridiaceae, Faecalibacterium prausnitzii*, and *Roseburia hominis*.^65–67^ The balance between *Firmicutes* and *Bacteroidetes* varies with obesity and the proportion of lean body mass.^68^ Enrichment of *Firmicutes* or a reduction in *Bacteroidetes* is considered an obesity-inducing trait that is commonly seen in obese children.^64^ The *Firmicutes:Bacteroidetes* ratio has become an important parameter in the evaluation of the relationship between gut microbiota, obesity and obesity-related disorders.^69^ Some studies have shown that exercise reduces the *Firmicutes:Bacteroidetes* ratio, which is beneficial to metabolic health.^64,66,70–72^ The decline in the *Firmicutes Bacteroidetes* ratio may be reflective of a lean phenotype and has been associated with adaptive metabolic consequences such as increased SCFA production, increased energy expenditure, and inhibited fat accumulation in adipose tissue.^73^ However, other articles have indicated that exercise increases this ratio.^74–76^

Most of these studies describe different taxonomic changes in the microbiota after exercise and often show different changes at the phylum level (e.g., changes in the *Firmicutes:Bacteroidetes* ratio) or in α and β diversity, which indicate species richness and diversity, respectively (Table 3). The effect of exercise on gut microbiota is conflicting. The reason for this difference is that exercise-induced alterations of the gut microbiota may depend on diet, species, animal age, obesity status, exercise modality, and exercise intensity. It is difficult to draw broad conclusions about how and to what extent exercise alters the gut microbiota of rodents and humans. Therefore, it is difficult to identify specific bacterial genera that produce a healthful response.

**Table 3.** Gut microbiota variations induced by exercise

Gut microbiota variations induced by exercise	Effects on Health	References
Butyrate-producing bacteria (*Bacteroidales S24-7, Clostridiaceae, Faecalibacterium prausnitzii, Roseburia hominis*)↑	Maintains intestinal homeostasis and health	^65–67^
*Firmicutes/Bacteroidetes*↓	Prevents obesity and increases metabolic capacity	^64,66,70–72^
*Firmicutes/Bacteroidetes*↑	Enhances aerobic capacity and reduces blood lactate concentration	^74–76^
Microbial diversity↑	Promotes metabolic health	^75,77^

(↑, increase; ↓, decrease)

#### 3.2.2 Exercise-induced changes in intestinal microbiota promote health

Exercise-induced changes in intestinal microbiota are associated with improved health status and impact the intestinal tract by increasing microbial diversity and functional metabolism.^77^ These changes could reverse conditions associated with metabolic diseases, inflammatory diseases, and neurological and behavioral disorders.^78,79^ In previous studies, increased microbial diversity was associated with health, such as cardiopulmonary adaptability and the gastrointestinal microbial metabolic spectrum.^80,81^ Exercise-induced microbial changes in rats were also associated with low insulin resistance, adipose tissue inflammation and better exercise tolerance.^82^ Moreover, microflora transplanted from exercising mice to nonexercising mice improved bacterial diversity and metabolite distribution and reduced colon inflammation.^83^ Exercise training resulted in a continuous decrease in systolic blood pressure and improved gut-brain axis function in spontaneously hypertensive rats, which was related to increased microbial α diversity, changes in β diversity and enrichment of beneficial bacteria.^84^

However, the effects of exercise on the microbiota were transient and reversible, and the exercise-induced changes in intestinal microbiota depended on the duration of exercise. Most of the bacterial groups and SCFAs that increased with exercise decreased during a subsequent 6-week sedentary period.^85^ Although high-intensity interval training increased insulin sensitivity and cardiovascular fitness, it did not alter the composition of the microbiome. This suggests that changes in the composition of the microbiome that occur with prolonged exercise training might be in response to changes in metabolic health rather than driven by exercise training-induced adaptations.^86^

Exercise has an independent effect on the gut microbiota, but longer or higher intensity aerobic training may be required to induce significant changes in bacterial taxa. Overall, exercise increases butyrate-producing intestinal microorganisms, enriches beneficial bacteria and improves the diversity of intestinal microorganisms, thereby preventing obesity, maintaining body health and slowing age-related diseases.

### 3.3 Drugs and intestinal microbes

Numerous studies have been undertaken to address the challenges of aging. Among them, studies using drug therapies to combat aging have grown exponentially over the past decade. The most promising drug interventions include rapamycin, metformin, resveratrol, acarbose, spermidine and aspirin, which can effectively delay the aging of model animals and the onset of various chronic diseases.^87,88^

Before oral drugs are absorbed by blood, they must be metabolized through the intestine. In this process, it is likely that the drug first reacts with the gut microbes colonizing the intestinal epithelium. The use of drugs may affect the composition of intestinal microorganisms in different ways. At least two modes of action have been proposed. The first mode of action is that drugs can lead to microbial transfer from other parts of the body to the intestine.^89^ For example, proton pump inhibitors can reduce the acid barrier of the stomach, allowing oral microbes to enter the intestine through the stomach and cause disordered microbial ecology.^90^ The second mode of action is thought to be dominant, in which drugs can change the intestinal microenvironment and directly affect bacterial growth.^89^ For example, metformin can promote the growth of bacteria that produce SCFAs in the intestine, and these bacteria ultimately contribute to the therapeutic effect of metformin in improving insulin resistance and glucose homeostasis.^91^ In addition, some drugs showing antibacterial activity can also inhibit the growth of specific bacteria.^92^ The therapeutic effect of some anti-aging drugs may also be related to changes in intestinal microorganisms (Table 4).

**Table 4.** Gut microbiota variations induced by drug treatment

Drug Treatment	Study Model	Gut Microbiota Variations Induced by Drug Treatment	Effects on Health
Rapamycin	*Drosophila melanogaster*^93,94^	Bacterial load↓ *Alphaproteobacteria*↓	Prolongs lifespan and promotes a healthy lifespan
	Mice^95^	Segmented filamentous bacteria↑	
Metformin	Rats^96,97^	*Lactobacillus, Verrucomicrobia*↑	
	Mice^97^	*Bacteroidetes, Verrucomicrobia, Akkermansia, Bacteroide*↑	
	Type 2 diabetes (T2D) patients^98,99^	*Intestinibacter*↓ *Escherichia, A. muciniphila, Akkermansia muciniphila*, butyrate-producing bacteria *(Butyrivibrio, Bifidobacterium bifidum, Megasphaera)*↑	
Resveratrol	Rats^100,101^	*Firmicutes/Bacteroidetes, Enterococcus faecalis*↓ Intestinal microbial diversity, *Lactobacillus, Bifidobacterium*↑	
	Mice^101–104^	*Firmicutes/Bacteroidetes, Firmicutes*↓ Intestinal microbial diversity, *Bacteroidetes, Lactobacillus, Bifidobacterium, Akkermansia*↑	
Acarbose	Mice^105^	*Bacteroides*↓ *Bifidobacterium, Lactobacillus*↑	
	T2D patients^106,107^	*Bacteroides*↓ *Bifidobacterium(Bifidobacterium longum), Eubacterium, Lactobacillus, Enterococcus faecalis* ↑	
Spermidine	Mice^108^	SCFA-producing bacteria *Lachnospiraceae NK4A136*↑	
Aspirin	Mice^109^	*Bifidobacterium, Lactobacillus*↑	

(↑, increase; ↓, decrease)

#### 3.3.1 Rapamycin

Rapamycin is a natural macrolide compound isolated from bacteria and a pharmacological inhibitor of TOR signaling. It can reduce the rate of aging and effectively improve age-related diseases.^110^ Rapamycin treatment has reportedly altered the number and structure of gut microbiota in flies and mice. The addition of rapamycin to food can significantly reduce the bacterial load of flies (40 days) and delay microbial expansion in the aging gut.^93^ It also reduces the level of *Alphaproteobacteria*, which is a group that has been previously recognized to be related to a decline in health and mortality in the elderly.^94^ In middle-aged mice, transient rapamycin treatment is also associated with microbial remodeling, including a dramatically increased prevalence of segmented filamentous bacteria in the small intestine.^95^ However, only a few studies have investigated the interaction between rapamycin and gut microbiota. Therefore, whether the change in gut microbiota plays a causal role in the beneficial effect of rapamycin treatment remains to be answered.

#### 3.3.2 Metformin

Metformin is the first choice for the treatment of T2D. In recent years, more studies have focused on the relationship between metformin and intestinal flora, indicating that metformin may, in part, exert its therapeutic effect through these microorganisms. In addition to treating T2D, metformin is also thought to be an anti-aging and health-improving drug. It was reported that metformin prolongs the lifespan of *C. elegans* by changing the metabolism of folic acid and methionine in microorganisms, but this effect was eliminated under sterile culture conditions.^111^ Recent studies have also proven that the therapeutic effect of metformin is related to changes in the intestinal flora.^96,98,99,112^

Metformin can change the composition of intestinal flora in HFD-induced obese mice and rats as well as in T2D patients, showing changes in the relative proportions of certain bacteria at different taxonomic levels. Interestingly, metformin can maintain intestinal barrier function, improve glucose homeostasis and exert hypoglycemic effects by affecting intestinal flora.^97^ Recent studies have shown that the incidence of colorectal cancer (CRC) in patients with T2D is high, and the occurrence of CRC is closely related to intestinal microflora. Metformin inhibits CRC in T2D patients by altering the abundance of gut microbiota or involving gut microbiota.^113^ Metformin can also inhibit microglial activation and neuroinflammation in the brain by regulating intestinal flora in obese mice and thus may be considered a promising candidate for the intervention of cognitive decline related to an obesity-induced imbalance of the gut microbiota.^114^ However, due to the complex composition of microbial communities, considerable differences in species, individuals and experimental design, the changes in gut microbes caused by metformin are not consistent, which presents great obstacles for us to better understand the relationship between metformin and gut microbes.

#### 3.3.3 Resveratrol

Resveratrol, a natural polyphenol with a wide range of pharmacological properties, is synthesized by plants in response to stress, injury, infection or ultraviolet radiation. As a multitargeted therapeutic agent for chronic diseases, it has potential in managing diabetes and cardiovascular and neurological diseases.^115^ Resveratrol and intestinal microorganisms have bidirectional interactions. Resveratrol can directly change the composition and diversity of intestinal microflora by inhibiting the growth of individual microbial species or causing population transfer.^116^ In turn, intestinal microflora can help the metabolism of resveratrol precursors to resveratrol and improve the bioavailability of resveratrol.^100^

Some beneficial effects of resveratrol are associated with gut microbes. Resveratrol improved the intestinal microflora imbalance caused by a HFD and played an anti-obesity role. The mechanisms included reducing the *Firmicutes:Bacteroidetes ratio* and promoting the diversity of intestinal microflora by inhibiting the growth of *Enterococcus faecalis* and increasing the abundance of *Lactobacillus* and *Bifidobacterium*.^117^ Other studies have also shown that resveratrol can play an anti-obesity role by improving intestinal microbial diversity and intestinal barrier function.^102,118^ Resveratrol attenuated trimethylamine-N-oxide-induced atherosclerosis by remodeling the gut microbiota and increasing the relative abundance of *Bacteroides, Lactobacillus, Bifidobacterium* and *Akkermansia* in mice.^103^ In addition, resveratrol can also improve the intestinal microflora under oxidative stress due to intestinal diseases, which makes it a promising potential drug for the treatment of IBD.^104^

#### 3.3.4 Acarbose

Acarbose is an antidiabetic drug used to treat T2D and is an oligosaccharide that reversibly inhibits intestinal α-glucosidase enzymes. In addition to diabetes, acarbose has proven to be beneficial in lowering the risk of cardiovascular disease and hypertension.^119^ Acarbose reproducibly modulated the composition of the microbiota and increased the concentration of SCFAs in mice, especially the abundance of propionate or butyrate. There was a correlation between fecal SCFAs and lifespan in mice, suggesting a role of the gut microbiota in the longevity-enhancing properties of acarbose.^120,121^ In another study, acarbose increased the abundance of *Bifidobacterium* and *Lactobacillus*, whereas the abundance of *Bacteroides* was decreased at the genus level.^105^ Consistent with a study in mice, acarbose treatment increased the abundance of *Bifidobacterium, Eubacterium*, and *Lactobacillus* and decreased the abundance of *Bacteroides* in T2D patients.^106^ Similarly, the gut microbiota *Bifidobacterium longum* and *Enterococcus faecalis* were increased significantly after 4 weeks of acarbose treatment in T2D patients.^107^

#### 3.3.5 Spermidine

Spermidine is a natural polyamine that can either be obtained orally from exogenous dietary sources or be produced by intestinal symbiotic bacteria and cellular biosynthesis. It has potential health promotion effects on aging and its comorbidities.^122^ A recent study found that spermidine treatment significantly altered the composition and function of the gut microbiota in obese mice, especially increasing the abundance of the SCFA-producing bacteria *Lachnospiraceae NK4A136*. These effects were lost after the depletion of the gut microbiota and restored by the transplantation of spermidine-treated microbiota into obese mice.^108^ This result indicated that in addition to the role of spermidine itself, the altered microbiota was also involved in spermidine-mediated anti-obesity effects. However, there are few studies on the relationship between spermidine and microbiota, and more studies are needed in the future.

#### 3.3.6 Aspirin

Aspirin is a historical antipyretic, analgesic and anti-inflammatory drug that can improve health and prolong lifespan in model organisms.^123^ After aspirin treatment, the bacterial composition of mice was changed, and the probiotics *Bifidobacterium* and *Lactobacillus* were enriched. However, *Bacillus sphaeroides* weakens the chemopreventive effect of aspirin on CRC by influencing the bioavailability of aspirin in mice.^124^ Coadministration of antibiotics can modulate the metabolism and pharmacokinetics of aspirin via suppression of metabolic activity of the gut microbiota in rats, which could potentiate the therapeutic potency of aspirin.^109^

## 4. Approaches for bacteriotherapy: probiotics, prebiotics and synbiotics

The human gut microbiota plays an important role in human health, and modulation of the gut microbiota may be used to treat and prevent an array of diseases. Bacteriotherapy includes three slightly different agents: probiotics, prebiotics, and synbiotics. They are appealing for the prevention and treatment of human medical disorders.^125^ The use of probiotics, prebiotics and synbiotics is a cost-effective and widely available intervention that may improve the homeostasis of gut microflora and prevent frailty and unhealthy aging.

Probiotics are defined as “live microorganisms that, when administered in adequate amounts, confer a health benefit on the host”.^126^ *Lactobacillus* and *Bifidobacterium* species are most commonly used. A large number of probiotics with anti-aging potential have been identified in various animal models. Some clinical studies have even proven the potential of some probiotics for the treatment of diseases such as intestinal diseases, metabolic diseases and neurological diseases.^127,128^ For example, probiotic intervention may reduce the risk of antibiotic-associated diarrhea by 51% with no apparent increase in the risk of side effects.^129^ *Bifidobacterium breve B-3* has potential as a functional food ingredient to reduce body fat in healthy preobese individuals.^130^ Consumption of *Lactobacillus plantarum C29*-fermented soybean for 12 weeks by elderly individuals with mild cognitive impairment showed improvements in cognitive functions.^131^

Prebiotics are defined as “a substrate that is selectively utilized by host microorganisms conferring a health benefit”, such as fructooligosaccharides, inulin and galactooligosaccharides.^132^ Prebiotics specifically stimulate the growth of endogenous microbial populations that are perceived to be beneficial to human health, such as *Bifidobacteria* and *Lactobacilli*.^133^ The presence of prebiotics in the diet may lead to numerous health benefits. Prebiotics can improve the microbiota and immunological changes associated with aging.^134^ A prebiotic intervention can reduce frailty levels in nursing home residents, especially in those with higher levels of frailty.^135,136^ Targeting the gut microbiome with prebiotics can overcome age-related anabolic resistance. Those who take prebiotic and protein supplements have a greater improvement in muscle strength than those who take protein supplements alone.^137^

Synbiotics are defined as “a mixture comprising live microorganisms and substrates selectively utilized by host microorganisms that confers a health benefit on the host”.^138^ They are usually combinations of probiotics and prebiotics. The synbiotics also shows some health benefits. The use of synbiotics comprising the probiotic *Bifidobacterium longum* and an inulin-based prebiotic can change the metabolism and composition of the gut microbiota in elderly people. The synbiotic increased the members of *Bifidobacteria, Actinobacteria*, and *Firmicutes* and was associated with increased butyrate production.^139^ Consumption of a synbiotic food for 6 weeks could affect the metabolic status of diabetic patients and had significant effects on serum insulin, high sensitivity C-reactive protein, uric acid and plasma total glutathione levels.^140^ In addition, synbiotics significantly decreased metabolic syndrome prevalence, several cardiovascular risk factors and markers of insulin resistance in elderly patients.^141^ However, due to the limited research on synbiotics, there is currently inadequate evidence to recommend synbiotic use to elderly people in general.^142^

Numerous studies have shown that the use of microbiome manipulation with probiotics, prebiotics, and synbiotics has health benefits, but microbial intervention may also have some potential health risks in the elderly population. Probiotics are the direct inoculation of live organisms into a host, potentially transforming these colonies from beneficial symbiotic bacteria to overt pathogens.^143^ While probiotics may be safe in healthy adults, their use has been associated with a higher risk of infection or morbidity in children, immunosuppressed individuals and critically ill individuals.^144^ Prebiotics are not systemically absorbed and have a limited side effect profile, and synbiotics carry the same risk as probiotics.^145^ Moreover, probiotic interventions may not be successful across the population, and their responses are driven by both host and microbiota characteristics. The choice of strains, dosage and duration of intervention can strongly influence the beneficial outcome.^146^ Colonization resistance is an important feature of the microbial community, which can protect us from pathologic infections. Simultaneously, the same mechanism may prevent probiotic colonization, and this colonization resistance may be human-specific. Antibiotic pretreatment may improve the colonization of probiotics, but the benefits of probiotics after antibiotics may be counteracted by compromised gut mucosal recovery.^147^ Therefore, the effects of probiotics on the host or its microflora may be variable.

The above studies suggest that modifying the gut microbiota of the elderly population by the intake of functional food as probiotics, prebiotics, or synbiotics may be an effective strategy to counteract natural aging. At the same time, these functional products may be suitable, affordable, and economical to most elderly people. However, their effects on health are complex, depending on individual populations and the duration of treatments. For efficacy and safety considerations, the development of probiotics, prebiotics and synbiotics for human health must take into account possible highly individual differences.

## 5. Conclusions and future perspectives

Aging is a major risk factor for almost all age-related diseases. Changes in the intestinal microflora with aging are related to the pathogenesis of age-related chronic diseases. Dietary intervention, exercise and drug therapy are currently the most studied anti-aging measures and can improve the intestinal microbial imbalance caused by aging and promote a healthier intestinal environment to achieve anti-aging effects. In addition, gut microbiota modification represents a promising intervention for anti-aging and aging-related diseases, such as the use of probiotics, prebiotics, and synbiotics. However, evidence of probiotic, prebiotic and synbiotic use in elderly people is in its infancy compared with other measures. A review of the current scientific literature can offer no direct conclusions regarding the efficacy of these measures.

Despite much research on these interventions, there are no firm conclusions about the benefits for human health. The reasons may be as follows: (1) Most of the relevant studies have been conducted in laboratory and animal models. These findings do not necessarily apply to humans directly. (2) Most clinical trials with humans are short-term and insufficient to understand long-term health effects. (3) Humans are quite different from each other in terms of sex, size, age, genetics, environment, lifestyle, and other factors. An anti-aging intervention that was found to help one person might not have the same effect on another. (4) Although many probiotics have proven strong safety profiles, we should still be careful to monitor their potential risks in different populations in the development of new probiotics. Therefore, future research needs to focus on addressing these issues to better understand the safety and efficacy of these anti-aging measures in humans. In addition, although much hope and investment are currently focused on drug development, the application of anti-aging drugs in humans still has a long way to go. It is important to note that sensible habits may be more effective at extending healthspan than taking a medication. This means eating healthy foods, exercising, drinking alcohol in moderation or not at all, not smoking, getting adequate sleep, and maintaining an active lifestyle.
